# A concise review on lipidomics analysis in biological samples

**DOI:** 10.5599/admet.913

**Published:** 2020-12-09

**Authors:** Ramani Venkata Addepalli, Ramesh Mullangi

**Affiliations:** 1 Palm Meadows, Kompally, Hyderabad-500100, India; 2 Laxai Life Sciences Pvt Ltd, MN Park, Genome Valley, Shamirpet, Hyderabad-500 078, India

**Keywords:** Lipids, LC-MS/MS, samples processing, bioanalysis

## Abstract

Lipids are a complex and critical heterogeneous molecular entity, playing an intricate and key role in understanding biological activities and disease processes. Lipidomics aims to quantitatively define the lipid classes, including their molecular species. The analysis of the biological tissues and fluids are challenging due to the extreme sample complexity and occurrence of the molecular species as isomers or isobars. This review documents the overview of lipidomics workflow, beginning from the approaches of sample preparation, various analytical techniques and emphasizing the state-of-the-art mass spectrometry either by shotgun or coupled with liquid chromatography. We have considered the latest ion mobility spectroscopy technologies to deal with the vast number of structural isomers, different imaging techniques. All these techniques have their pitfalls and we have discussed how to circumvent them after reviewing the power of each technique with examples..

## Introduction

Lipids are a complex and critical heterogeneous molecular entity, playing an intricate and key role in many biological functions such as acting as structural scaffold for the cell membrane, serving as energy storage and participating in signaling pathways. Lipid classification system is spearheaded by LIPID MAPS^®^ Lipidomics Gateway (https://www.lipidmaps.org/). Lipids can be divided into eight main categories: fatty acyls (FA), glycerolipids (GL), glycerophospholipids (GP), sphingolipids (SP), sterol lipids (ST), prenol lipids (PR), saccharolipids (SL) and polyketides (PK) [1] (Table 1). Each category can be further classified into different lipid classes and subclasses, based on the number of carbon atoms and double bonds, the branching of the hydrocarbon chain, the position and orientation of double bonds, the addition of polar groups such as choline, inositol and ethanolamine; and glycosylation [2]. The LIPID MAPS structure database currently records 45245 lipid structures (as of 12 March 2020) (https://www.lipidmaps.org/). The complete lipid profile within a cell, tissue or organism is known as a lipidome and is a branch of the “metabolome”. Lipidomics is a discipline that studies the characteristics of lipid and to unravel the complex interactions of lipid metabolites in a biological system.

A specific alteration in the lipidome, provides potential insights into perturbed pathways, physiological processes and ultimately stages of diseases. Clinical lipidomics was defined “as a new integrative biomedicine to discover the correlation and regulation between a large scale of lipid elements measured and analyzed in liquid biopsies from patients with those patient phenomes and clinical phenotypes” [3]. To cite few examples, lysophosphatidic acid stimulates cell proliferation, migration and survival by acting on its cognate G-protein-coupled receptors in cancer cells [4]. Alzheimer’s and Parkinson’s diseases have been linked with aberrant cholesterol and abnormal glycolipid metabolism, respectively [5]. Lipoprotein abnormalities are associated with Type II diabetes with increased triglycerides (TG) and very low density lipoprotein (VLDL) levels, whereas decreased apolipoprotein E/VLDL-TG ratio in ischemic heart disease [6]. Depending on associated underlying pathways, these lipids serve as a potential biomarkers and a diagnostic tool. For instance, GL, SP, linoleic acid, cholesterol serve as a biomarker for Alzheimer’s; lycophosphatidylinositol and prostaglandins (PG) for Parkinson’s; ceramides, sphingomyelin for Type II diabetes mellitus and glycosphingolipid for obesity [7].

**Figure 1.**
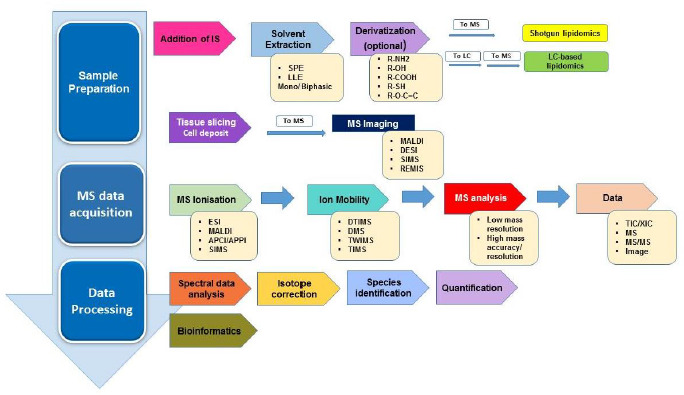
Typical work-flow of lipidomics analysis in biological samples

Owing to the diversification of the lipid classes with various combinations of polar head groups, fatty acyl chains, backbone structures, identification and characterization of lipids is very complicated. Aggravating to this is its extensive expansion of the applications. The prime objective of lipidomics is to attain full coverage of precise structural analysis, accurate quantification and understanding its dynamics. The available analytical techniques are broadly categorized into three groups, namely global lipidomic analysis, targeted lipidomic analysis and novel lipid discovery. The global lipidomics deals with identification, quantification hundreds to thousands of cellular lipids via a high throughput basis. Shotgun lipidomics-based platforms are extensively used in this category to analyze diverse pathways and networks associated with lipid metabolism, trafficking, and homeostasis. As an extension, mapping techniques have also been used to study the spatial and temporal relationships of lipids. Targeted lipidomic analysis also deals with the identification but with one or a few lipid classes of interest using LC-MS (liquid chromatography coupled to mass spectrometry) and LC-MS/MS (liquid chromatography coupled to tandem mass spectrometry) based methods. While the novel lipid discovery deal with novel lipid classes and molecular species using LC coupled with MS with different enrichment technologies. In this review, we are providing an overview of the current understanding of lipid analysis taking into account the workflow, methodologies, technical characteristics and bottlenecks. We have also listed important papers and reviews that cover most of the aspects of lipidomics. We hope that this review will act as a bridge for biomedical and pharmacological research to generate novel approaches to disease diagnostics. A typical work flow of lipidomic analysis in biological samples is as shown Figure 1.

## Sample preparation

### Sample processing and storage

The most important and vital step in any bioanalytical work is sample processing. The biological samples can be solid in nature (e.g., tissues or cells) or comprise of highly complex biofluids (e.g., plasma, serum, urine or cyst fluid). It is laborious to handle solid samples as it involves additional disruption step. It is highly advisable to process the samples immediately, especially with the whole blood samples [8] or at least flash frozen as lipid samples exhibit substantial circadian variations. It is a well-known fact that the plasma concentration of lysophosphatidylcholine (LPC) or lysophosphatidic acid (LPA) increases when left at room temperature for a long period [9]. On the other hand, cardiolipin (CL) during the freezing process hydrolyses into monolysocardiolipin [10] and methanolic samples of lysophospholipid regioisomers isomerize at temperatures above 20 °C and at pH > 6.0 [11]. Freeze-thaw is another problem with specific lipid classes, aliquoting the samples before freezing will minimize its effect, example sphingosine, polyunsaturated fatty acids and eicosanoids [12,13]. Yet another problem is lipid oxidation and is a major concern with polyunsaturated fatty acid moieties, oxidized lipids and eicosanoids, special care should be exercised in handling, as these end products also contribute to both physiological and pathophysiological processes [13]. Storage of plasma/serum samples and their extracts under an inert gas (e.g., argon) may also limit oxidation. Antioxidants like butylhydroxytoluene (BHT) have been in use, the used concentrations and the time-point of its addition vary in the literature [14]. The efficacy and protocols for the use of antioxidants should be verified. However, oxidation is not a problem in quantifying abundant lipid classes (e.g., phospholipids, sphingolipids and TGs) [13]. It is advisable to exercise caution while handling samples with each class of lipids.

Biofluids, such as urine, serum, plasma and whole blood are frequently used as these do not require any homogenization but while working with the lipids from a piece of tissue or ruptured cells (e.g., organelles), sample disruption nonetheless has a significant impact on the end results of a process to make it accessible to extraction solvents. Widely used mechanical methods are liquid based homogenization (Potter-Elvehjem homogenizer, ULTRA-TURRAX), bead bearing (Bead Ruptor 24, Elite Bead Mill Homogenizer) and crushing of liquid-nitrogen-frozen tissue by pestle and mortar [15]. Latter approach is very slow as it is performed manually. Ulmer *et al*. (2017) have demonstrated the use of zirconium oxide or ceramic bead for softer tissues and stainless steel bead for muscle and harder tissues [16].

### Lipid extraction protocols

There are numerous published lipid extraction methods that can also be automated for high-throughput analysis [17-19]. This process reduces the complexity of the sample by enriching the analytes of interest and getting rid of any unwanted contaminants to mass spectrometer. The extraction principles are one/two-phase liquid-liquid extraction (LLE), solid-phase extraction (SPE) with varying parameters of extraction like temperatures, sample/solvent ratio, re-extractions, use of sonication, vortexing, extraction under inert gasses to enhance the lipid recovery. However, this process is accountable for the artifacts in lipid identification and inconsistencies in quantification.

The sample preparation technique most widely used in lipidomics is LLE. The Folch protocol and the Bligh and Dyer protocol both rely on a ternary mixture of chloroform, methanol and water [17,18]. One-phase extraction (OPE) method was proposed by Pellegrino *et al*. (2014) which is a mixture of methanol/chloroform/methyl *tert*-butyl ether (MMC) in the ratio 1.33/1/1 (v/v/v) [20]. Matyash *et al*. (2008) and Baker *et al*. (2001) proposed methyl-*tert*-butyl ether high-throughput lipidomics and acidified butanol extraction procedure for lysophosphatidic acid from biological samples [19,21]. It is well known fact that solubility can be enhanced by addition of some acid to the organic layer containing anionic lipids e.g. phosphatidic acids (PA), phosphatidylinositols (PI) or sphingosine-1-phosphate (S1P) to increase the extraction efficiency. We recommend to check the website http://cyberlipid.gerli.com/techniques-of-analysis/extraction-handling-of-extracts/ for guidance on extraction protocols.

### Derivatization

Chemical derivatization has substantially improved the shortcomings of mass spectrometric based shotgun lipidomics, liquid chromatography and more so in gas chromatography based applications. Though it is an additional step, it offers several advantages, it enhances the ionization efficiency, selectively introduce a fragment which can be used in precursor ion or neutral loss scans, masks the functional groups that contaminate the mass spectrometer (MS), often encountered with the lipids containing phosphates and most importantly, it helps in differential quantitation by selectively introducing an isotopic label. All these strategies were used by different scientists. The plasma samples for 7-oxocholesterol and 5,6-epoxycholesterol were chemically derivatized with Girard’s reagent P to increase the ionization efficiency of the intermediate metabolites in the patients suffering from lysosomal storage disorders [22]. Wang *et al*. (2017) quantitatively analyzed phosphatidylglycerols and bis(monoacylglycero) phosphates by diazomethane-based methylation of phosphate group to introduces class-specific fragments into the MS/MS spectra [23]. To prevent the contamination of the MS, Clark *et al*. (2011) methylated the phosphate groups to quantify phosphatidyl inositol phosphates [24]. Last strategy was adopted by Lee *et* *al*. (2017) who introduced a stable isotope-labeled methylation into one sample to enhanced detection and quantification of targeted phospholipids [25]. Chemical derivatization with N-[4-(amino methyl) phenyl] pyridinium prevented the molecular masses overlapping signals between ceramides and branched fatty acid esters of hydroxy fatty acids (FAHFAs) to a new region thereby reducing the false results of FAHFAs [26]. New chemical derivatization approaches were employed on targeted lipids by Zhao *et* *al*. (2020) [27]. However, there are lot of shortcomings in derivatization procedures and such procedures are expected to be rapid, high yielding, specific, in-situ, or biocompatible in order to meet the needs of studies.

### Internal standards

Internal standards are required to quantify bonafide concentrations of an analyte of interest in MS analysis. Both the internal standard and analyte are analyzed simultaneously to compensate for inherent variations in sample processing during the entire process of sample preparation and analysis (e.g., variations in lipid extraction efficacy, processing losses, matrix effects i.e., ionization suppression or enhancement). In an ideal scenario, both the internal standard and the analyte should be structurally similar and have a comparable MS/MS fragmentation pattern. So, a clear understanding of the types, concentration and characteristics of internal standards to be used for accurate quantification of lipid species, subclasses, and classes is of utmost importance. Ideally a stable isotope of the analyte of interest, if commercially available, should be used as an internal standard for quantitative analysis, provided quantification is limited to one or a limited number of species [28,29].

Internal standard mixes in lipidomic analysis approach is very common to biomarker discovery. Few researchers are of the opinion that one internal standard per lipid class is enough for quantitation [29], because ionization of lipids is largely dependent on the class specific head group and not so much on the fatty acyl chains [30]. Whereas, few others have contradictory report about the influence of fatty acyl chain length and unsaturation on ionization efficiency [30], it is highly advisable to extensively evaluate on case to case basis on this issue. The commercially available internal standard mixes containing isotope-labeled species include a wide range of acyl chain length and degree of saturation [31]. At least two internal standards are required to correct the effects of differential fragmentation kinetics and thermodynamics [32]. However, very few reliable internal standards with adequate chemical purity and exactly known lipid content are commercially available for a limited number of lipid subclasses and fatty acid compositions.

An internal standard cocktail has been developed for the LIPID MAPS Consortium which is commercially available as SPLASH^®^ LIPIDOMIX^®^ from Avanti Polar Lipids for quantitative mass spectrometry analysis (https://avantilipids.com/product/330707, 2018). This is specifically designed to complement human plasma lipid analysis using LC-MS/MS platforms. This contains uncommon chain-length sphingoid bases (C17) for sphingosine (So), sphinganine (Sa) and their 1-phosphates (S1P and Sa1P) and C12:0 fatty acid analogs of Ceramide (Cer) ceramide 1-phosphate (Cer1P), sphingomyelins (SM), simple mono- and dihexosylceramides (HexCer and diHexCer). It also includes sulfatides, 1-deoxy- and 1-(deoxymethyl)-sphingoids, glycerol-phospholipids, phosphatidylinositol-bisphosphate, sterols and neutral lipids (https://avantilipids.com/product/330707, 2018).

To get consistent and robust results in a high throughput clinical analysis, the use of commercially available ready-made internal standard mixes with exactly known concentrations has an added advantage. The lipidomics community should encourage the development of novel comprehensive and easily available isotope labeled internal standard mixes.

## Analytical techniques for the study of lipids

### Shotgun Lipidomics

Identification and quantification of a cellular lipidomics directly from organic extracts of biological samples based on chemical and physical properties of lipid classes, subclasses, and individual molecular species are the prime aim of shotgun lipidomics [33]. This concept was based on a simple technique of a conventional loop injection, using a syringe coupled with tandem quadrupole mass spectrometry analysis of precursor masses and fragment mass, refraining from chromatographic separation [33,34]. This evolved into static nano-ESI source [29] without syringe pump; resulting in higher ionization efficiencies. Han *et al*. had used intra-source separation, favoring the ionization (i.e., positive- and negative-ion ionization) of selected lipid classes through solvent additives and subsequently using precursor ion and neutral loss scans of polar head group, resulting in fatty acid moieties [34,35]. Sufficient quantitation was achieved by addition of one internal standard per lipid class [36] as ionization largely dependents on specific head group and not so much on the fatty acyl chains [37]. Though some scientists contradict that fatty acyl chain length and unsaturation influence ionization efficiency [38]. Another direct infusion methodology adopted by Guan *et al*. (2006) was coupling triple quadrupole analyzer in multiple reactions monitoring (MRM) mode with syringe pump [39]. This was used to quantify the major lipid components in the lipid extract but critically lies in the knowledge of anticipated precursor and product ions. Although this technique is simple, ease of management and less expensive, due to the continuous infusion cross-contamination, isobaric overlaps of the M + 2 isotope with the monoisotopic peak of the compound are major limitations.

To overcome the contamination problem from the carryover of previous samples, multi-dimensional mass spectrometry based shotgun lipidomics (MDMS-SL) was used to study the composition of lipid structures [37]. Using this technique differential intra-source separation properties with various additives like Li^+^ or NH4^+^ or Na^+^, resulted in unique fragments for each lipid class. Nanoelectrospray ionization was integrated with chip-based nano-ESI platform (Advion NanoMat^®^) for better sensitivity and high reproducibility of sample infusion. Quantitative applications of various classes of glycerophospholipids, sphingolipids and glycerolipids were studied using this technique. However, this devise is not cost effective and has limited stability of the electrospray due to clogging. Moreover, samples were subjected to analysis sequentially, by charge separation (e.g., deprotonation) or adduct formation (e.g., protonation, alkaline metal adduct ion, halogen adduct ion) making the data handling difficult where specialized software packages are required, which will be discussed later in this article. Other disadvantage being separation of isobaric lipids is not possible. In an unique application, differential mobility spectrometry (DMS) separation was combined with ionization by electrospray ionization (ESI) to separate many isobaric and isomeric lipids, which is also not fully evolved [40]. Nonetheless, this was a quick and reliable technique. Detection of unexpected lipid species and vulnerability for overlapping isobaric compounds is the major limitation of this method.

Automation was brought about by replacing syringe pump with HPLC (high-performance liquid chromatography) and coupling with triple quadrupole analyzer. The HPLC pump runs at micro-flow rates and the auto-sampler injects samples into the flow directly delivering into the ESI (electro-spray ionization) source sans the column [41]. Robustness and high automation made data acquisition simpler, as multiple precursor ions and constant neutral loss scans could be achieved by concentrated sample pulse and multiple injections. Multiple standard curves were achieved in quantitation of lipids (different fatty acyl chain lengths and degrees of unsaturation) by standard addition method and one internal standard [42]. This method was further applied on various subclasses of glycerophospholipids, sphingolipids and sterols [42]. However, low resolution direct infusion technologies suffer from general dogmas of limitations, such as ion suppression, ambiguous identification of isobaric/isomeric lipid species, and ion source-generated artifacts, hindering the applications to low-abundance lipid species, particularly in less ionizable or isomers bearing identical fragmentation patterns. One such example, diacyl and acyl-alkyl glycerophospholipids, isobaric phosphatidylcholines with odd-carbon-numbered fatty acids from plasmalogens [43].

To deal with this, shotgun lipidomics has evolved into a myriad of multi-dimensional strategies for molecular lipid characterization by coupling with high resolution mass spectrometers (HRMS) like quadrupole time of ﬂight (Q-ToF) or Orbitrap or Fourier transform ion cyclotron resonance (FT-ICR) [44-46]. HRMS measures exact mass, time-of-flight MS (ToF-MS), Orbitrap MS and FT-ICR-MS deliver mass resolutions of 60,000, 240,000 and more than 1,000,000, respectively. HRMS is especially useful because of their rapid acquisition of MS/MS spectra, higher mass resolution and optional MS^n^ fragmentation, enabling fingerprint studies without prior separation and eliminating the possibilities of false-positive identification. Choi *et al*. (2014) used untargeted lipid analysis to achieve detection of nine lipids in plasma after rosuvastatin treatment to explicate the side effects of the drug by using QToF-MS [46]. Further, this facilitates data-independent acquisition (DIA) MS/MS^ALL^, which has a wide mass range of spectral coverage to perform qualitative analysis. Gao *et al*. were pragmatic in quantification of cardiolipin in mitochondrial preparations [47]. Major shortcoming of MS/MS^ALL^ is that it leads to eventual loss of information on precursor-fragment relationships, complicating the identification of lipids. Almeida *et al*. (2014) utilized full fragmentation power of an Orbitrap Fusion Tribrid and sequentially acquired higher-energy collisional induced dissociation (HCD) and collisional induced dissociation (CID) and ion trap mass spectrometry (ITMS3) to structurally characterize molecular glycerophospholipid species [44]. Using this novel high confidence filtering strategy, 311 lipid species circumventing 20 lipid classes and identification of 202 distinct molecular glycerophospholipid species in mouse cerebellum and hippocampus was achieved. Flaherty *et al*. (2019) evaluated the levels of multiple lipid species in bone marrow-derived macrophages using triple quadrupole/ion trap mass spectrometer [48].

### Liquid-chromatography-mass spectrometry

Shotgun approach is extensively used because of its simple, rapid and ease of handling the crude lipid extracts. But the major pitfall of shotgun is highly convoluted spectra due to matrix interference (ion suppression and ion enhancement effects), functional group modification, and occurrence of the molecular species as isomers or isobars throws greatest challenges to separation scientists. This requires good analytical separation platforms i.e., LC-MS to reduce the above drawbacks. Further, coelution of lipids had replaced conventional HPLC systems with faster run times, highly efficient separation owing to the higher backpressure allowance, ultra-high-pressure liquid chromatography (UHPLC). Integrating UHPLC with ToF-MS (UHPLC/Q-ToF-MS) is powerful tool, which enables high resolution chromatographic separation coupled with structure elucidation and identification of fragmentation patterns of the comprehensive nontargeted lipid analysis [49].

The most important separation techniques used in lipidomics are RPLC (reverse phased liquid chromatography), NPLC (normal phase liquid chromatography) and HILIC (hydrophilic interaction liquid chromatography). Other separation techniques are also occasionally used include non-aqueous RPLC (NARP) [50], silver-ion RPLC [51], chiral LC [52] and supercritical fluid chromatography (SFC) [53]. Each technique was used by different group of scientists to resolve the complexity of the lipidome. Firstly, non-aqueous RPLC was use by Lin *et al*. (1997) the separation of molecular species of 45 synthetic triacylglycerols and diacylglycerols, due to the advent of new column technologies this use is limited [50]. Secondly, non-aqueous reversed-phase (NARP) and silver-ion high-performance liquid chromatography with APCI-MS and GC/FID detection were used for the characterization of fatty acids and triglycerides composition in complex samples of animal fats [51]. Thirdly, complex mixtures of regioisomeric and enantiomeric eicosanoids (hydroxy and hydroperoxy fatty acids) have been resolved using chiral lipidomics approach using electron capture atmospheric pressure chemical ionization/mass spectrometry [52]. Finally, SFC coupled with Orbitrap mass spectrometry based lipidomics platform was used to identify diverse lipid molecular species [53].

#### Reversed-phase LC

RPLC is most widely used and separations are based on lipophilicity of interacting components, e.g. shorter carbon chains and polyunsaturated analogs being more polar elute earlier as compared to longer carbon chains and saturated acyl structures, respectively. Before 2004, long narrow (100-250 mm), normal bore (2-4.6 mm I.D.) columns with higher particle size (3.5-5 μm) were used. With the advent of UHPLC columns with 2 μm particle size were in use with higher flow rates and are resulting in better resolution. Further, to decrease the diffusional mass transfer path, operating at higher speeds and lower back pressures, “fused core” technology was used with 2.6-2.8 μm particles with a 0.35-0.5 μm porous shell fused to a solid core [54]. C18, a core-shell column, showed superior performance in case of chromatographic peak characteristics (plate number, number of detected lipid features) [55]. Few applications focused on miniaturization of lipidomics analyses, employing capillary (50-650 × 0.15-0.32 mm I.D.; 1.7-5 μm) and nanobore (50-170 × 0.075 mm I.D.; 3-5 μm) columns; as these offer higher sensitivity and smaller sample requirements at lower flow-rates of 0.3-10 μL/min [56].

In lipidomics, C18 or C8-modified sorbents based RPLC columns of short length (50-150 mm; typically, 100 mm) microbore (1.0-2.1 mm I.D) with particle size of sub-2 μm or 2.6-2.8 μm (fused-core) are majorly used. Examples are the Acquity UPLC BEH C18, Zorbax Eclipse XDB-C18, Acquity UPLC HSS T3, Acquity UPLC BEH C8, Kinetex C18 and Ascentis Express C8 columns. These columns can be used at a flowrate of 0.1-0.5 mL/min and 40-55°C temperatures. Apart from these, C30 stationary phase are also used, though their use is limited in untargeted profiling of lipidome, but its potential has been demonstrated in separation of phospholipids [57]. The geometric and positional isomers of structurally related lipids having conjugated double bonds can be separated by polymeric C30 stationary phase; this is attributed to phase thickness [58]. A comprehensive untargeted lipidomic analysis using core–shell C30 particle column has been demonstrated by Narváez-Rivas and Zhang [59]. These columns showed an excellent resolution of triglyceride regioisomers, which only differed in fatty acid positions (*sn*-1, 2 or 3) on the glycerol [60]. Few examples of C30 columns are Acclaim™ C30, Accucore™ C30, HALO C30. Along these column chemistries, achieved good LC separation and detection of lipids by RPLC; a mixture of water with or without different combinations of mobile phase additives of ammonium formate or acetate (5-10 mM), formic or acetic acid (0.05-0.1%) and organic solvent(s) like acetonitrile, methanol, isopropanol (IPA) or tetrahydrofuran (THF) have to be used. RPLC has few limitations in separating phospholipids.

#### Normal-phase LC

Separation of phospholipids with RPLC is not very efficient, so NPLC is used even though analysis time is more because of long length (100-250 mm; typically, 150 mm) though having microbore (2 mm I.D) with 3-5 μm particle size and separates lipids based on their polar functional group [56]. Few examples are Luna 3 μm silica, LiChrospher Si 60, Betasil silica-100, and Nucleosil 100-5 OH. These columns are operated at a flow-rate of 0.1-0.5 mL/min but with higher flow-rate (1.0 mL/min) split mode should be used and maintained at temperatures of 20-35 °C. Mobile phase employed over here are highly non-polar solvents with low ionization capacity like heptane, propan-1-ol, methyl *ter*-butyl ether, chloroform, ethanol and methanol. Different proportions of these solvents can be used to get weak to strong mobile phases. At times, additives like 0.5% NH_4_OH; 5-15 mM ammonium acetate, ammonium formate, diethylamine, formic acid, or small amounts of water (0.5-3%) are added to get adequate separation of lipidome.

#### Hydrophilic interaction chromatography (HILIC)

HILIC technique offers the benefits of both normal-phase and reverse-phase in lipid separations. HILIC columns exhibited extraordinary separation of lipid classes based on head group composition because of its hydrophilic properties; while using the same RPLC mobile phases to improve ionization efficiency and reproducibility of fatty acid chain length, degree of saturation and double bond position [61].

Lipid separations under HILIC conditions are usually conducted on shorter length (100-150 mm) micro-bore (2 mm I.D.) columns with 1.7-5 μm particle size, such as Atlantis HILIC silica, Acquity UPLC BEH HILIC, Nucleosil 100-5 OH and Spherisorb Si. These columns are operated at a flow-rate of 0.1-1 mL/min and maintained at temperatures of 25-40 °C. The analysis time is typically in the range of 15-60 min. Weak and strong mobile phases are used with high proportion of acetonitrile and water respectively, with methanol and IPA. Additives used are 0.1-0.2 % formic acid, 5-10 mM ammonium acetate, 20 mM ammonium formate, or 10 mM NH_4_OH. It has been demonstrated that HILIC can be used as an alternate system in a mix-mode with reverse phase liquid chromatography in the analysis of complex lipids [56,57].

Extreme diversity and challenges are faced in the analysis of complex lipids (isobars, regioisomers, ether-linkage or head group modification). To overcome this, many scientists have combined two liquid chromatography platforms to reduce the sample complexity, analysis times and elevated linear dynamic range [61]. HILIC and C30 reversed-phase chromatography (C30RP) coupled to high resolution mass spectrometry was used to analyse modified class of acylphosphatidylglycerol (acyl PG) in corn roots by HILIC, and further resolution of the isomers was enhanced using C30 RP chromatography [62]. Rampler *et* *al*. (2018) had used orthogonal HILIC and RP separations in parallel and the effluents of both columns were combined prior to high-resolution MS detection, to achieve full separation in one analytical run [63].

#### Long micropillar array columns (μPAC)

This unique technology was developed by a lithographic etching process to create a perfectly ordered separation bed on a silicon chip. Freestanding nature of the pillar offers several advantages compared to conventional columns by elimination of heterogeneous flow paths in the separation bed, thereby low backpressure, high resolution and high sensitivity. Sandra *et al*. (2017) has demonstrated inter and intra class separation and resolving isomeric lipids [64]. The blood plasma lipid differentiation of lyso-glycerophospholids (Lyso- GPs) and monoglycerides (MGs) from the glycerophospholipids (GPs), sphingolipids (SPs) and diglycerides (DGs) was studied [64]. This is commercially available with PharmaFluidics (https://www.pharmafluidics.com).

### MALDI and MALDI-ToF mass spectrometry

MALDI-ToF is an ideal complementary tool for shotgun lipidomic experiments since late 1990, because of its excellent sensitivity, high tolerance against salts, sample impurities, instrument robustness and freedom from crossover sample contamination [65]. Imaging lipids, mapping the distribution of various lipids in different tissues have been successfully carried out by many researchers using this technique which will be discussed later in this review. However, right choice of matrix plays a pivotal role in MALDI-ToF, for example free fatty acids analysis is difficult with standard matrixes, 2,5-dihydroxy benzoic acid and alfa-cyano-4-hydroxy cinnamic acid, due to the signal and matrix overlap. Instead basic matrixes like 9-amino-acridine and 2-mercaptobenzothiazole have been in use. Schiller *et al*. (1999) had comprehended an article on various conditions and matrices used in this technique [66]. So, MALDI-ToF provides a fast, easy, and useful tool for profiling complex lipid mixture, microbial lipids such as lipid A [67] and phosphatidylinositol mannosides [68]. MALDI-MS can be very easily coupled with thin-layer chromatography (TLC) allowing the spatially resolved screening of the entire TLC plate and the detection of lipids with a higher sensitivity and nondestructively in comparison with IR lasers and UV lasers [69]. Major setback of this method is in the quantitative analysis, due to lack of reproducibility, lack of universal matrix and interference of chemical background noise especially in low mass regions. A combination of high-energy collision-induced dissociation (CID) and prompt ion detection characteristic for MALDI ToF/ToF MS/MS has a unique feature of remote fragmentation of lipids at the level of fatty acyl *sn* position and double bond location, making the structural analysis easy [70]. Pittenauer *et al*. (2011) have illustrated many applications using this [71].

### Ion Mobility Spectrometry

Separation of isomeric and isobaric species in complex biological samples is a major challenge in HRMS; either shotgun MS or coupled with liquid chromatography MS. Ion mobility separates ions based on their differential mobility (size, shape, charge) through an inert gas (typically helium, argon or nitrogen) under the influence of an electric field [72,73]. The IM-MS is a strong synergy between these two techniques because of their ability to ascertain complementary information about gas-phase ions. Three-dimensional separations are achieved by a range of front-end techniques, IMS and MS providing fast measurements, providing new insights into lipid biology [74]. The front-end analytical separations include gas chromatography, supercritical fluid chromatography, liquid chromatography, solid-phase extractions, capillary electrophoresis, field asymmetric ion mobility spectrometry and microfluidic devices [74]. Whereas MS includes time-of-flight mass spectrometers (ToF-MS), quadrupole mass spectrometers (qMS), ion trap mass spectrometers (IT-MS), Fourier transform mass spectrometers (FTMS) and magnetic sector mass spectrometer. The major ionization sources are electrospray, MALDI and Laser spray ionization. Paglia *et al*. (2014, 2015) have done extensive work in this field and provide incredibly detailed protocols on various IMS-coupled mass spectrometry methods [75,76].

#### Ion Mobility Spectroscopy technologies

There are four commercially available IMS-MS technologies that have been utilized for lipidomics analysis: (i) Drift Time Ion Mobility Spectrometry (DTIMS) - here packets of ions are injected into a drift tube filled with an inert buffer gas. Under the influence of a weak electric field, ions are separated by charge, size, and shape, developed by Agilent Technologies [77] (ii) Field Asymmetric Ion Mobility Spectrometry/Differential Mobility Spectrometry (FAIMS/DMS) - an asymmetric waveform is applied to two cylindrical plates such that ions experience alternating high and low electric fields. Ions traverse the region between the plates moving in a perpendicular direction to the buffer gas and with the influence of a DC potential, termed the compensation voltage (CV). Only selected ions at a given CV will make it through the drift region, this approach is provided by Sciex [78] (iii) Travelling Wave Ion Mobility Spectrometry (TWIMS) - an alternating phase radio-frequency (RF) potential is applied to a series of stacked ring ion guides (SRIGs). Ions are pushed through the drift region with a traveling potential wave and become mobility separated as higher mobility ions are able to ‘roll-over’ the traveling waves generated and exit the SRIG region, developed by Waters [79] and (iv) Trapped Ion Mobility Spectrometry (TIMS), this uses an electric field to hold ions stationary against a moving gas, so that the drift force is compensated by the electric field and ion packages are separated based on their respective ion mobilities and process called parallel accumulation serial fragmentation or PASEF, developed by Bruker [80]. A detailed description of the theoretical concepts we refer the readers to reviews in this field [73,81].

#### Improved lipid identification by IM-MS

Identification of lipids has been done by accurate mass match with online databases such as LIPID MAPS or LipidBlast, but it provides only molecular formula. This is inconclusive as number of species belonging to different lipid classes has same molecular formula. The physicochemical characteristics of the compounds are required to allow a more accurate identification. This allows the calculation of the collision cross section (CCS), a four-dimensional orthogonal (retention time, *m/z*, ion mobility, intensity) physicochemical measure that can be used, together with accurate mass, fragmentation information and retention time (Rt) to increase the confidence of lipid identification in milliseconds. It is a known fact that, saturated lipids bearing acyl chains are extended in the electric field and have larger CCS values as compared to the unsaturated bonds with bent structure in the acyl chain. The CCS values are more influenced by the structural characteristics of compounds than the degree of saturation [75,82-84]. This was further correlated with the CCS values of FAs and PCs with both the lipid chain length and the degree of unsaturation. Many scientists have determined the CCS values to cover the lipidome of complex biological matrices [83,84]. Catherine et al. compiled 1856 lipid CCS values from plasma, liver and cancer cells with high quality of ^TIMS^CCS values [85]. Zhou *et al*. (2017) developed a support vector regression model using bioinformatic approaches and set of molecular descriptors, earnestly describing the subtle structural differences for lipids on SMILES structures [86]. These in silico LipidCCS values are independent data and are externally validated from the experimentally (TWIMS and DTIMS) determined values. Lipid CCS Predictor offers (i) prediction of lipid CCS values; (ii) LipidCCS database search and (iii) lipid match and identification [84]. The Lipid CCS database approximately contains over 15,000 lipids with over 60,000 corresponding CCS values determined experimentally or predicted in silico. CCS evaluation an additional identifying factor to improve data interpretation and enhance lipid identification in untargeted workflows [75]. However, validation of CCS values is restricted to the limited number of commercially available lipid standards and can only be used as an in silico CCS prediction to improve identification efficiency in lipidomics.

#### Applications/Isomer separation

**Figure 2.**
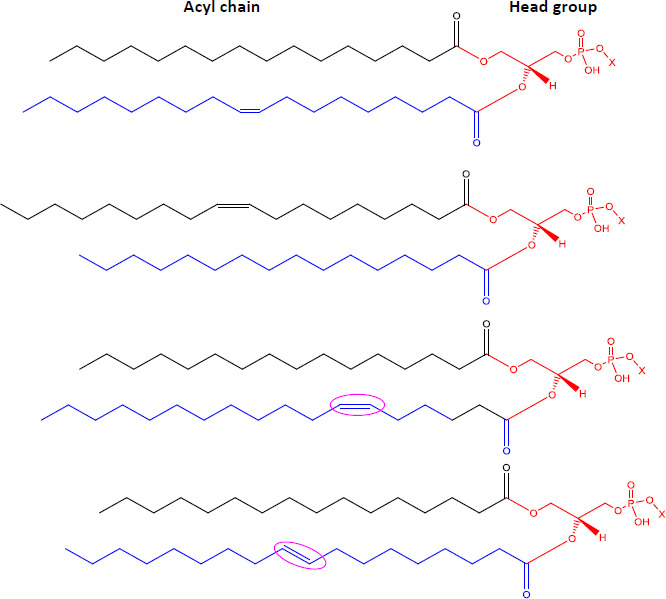
Isomeric structures of glycerophospholipids [Polar head group indicated as ‘x’ can be glycerol, choline, ethanolamine, inositol, or serine and two fatty acyl groups at sn1 (16:0) and sn2 (18:1 (9Z)) position forming the GP tail. Three types of isomers are represented - regioisomer presenting the fatty acyl chains in inverted positions; a positional isomer, containing the double bond in a different position (C6 instead of C9), and a geometric isomer, whose double bond is in trans (E) conformation]

Ion mobility system is exceptionally well-suited for untargeted lipidomics due to high-resolution, high-throughput and structural elucidation capabilities. Notable separation occurs in ion mobility (IM) before fragmentation (MS), as such product ions are mobility-aligned to corresponding precursor ions thereby improving interpretation of product ion spectra. Also, identical product ions derived from different precursor ions are proportionately assigned to their precursors, enhancing the low-abundance species detection [87]. One of the major confrontation in lipid analysis is isomer separation [88], with regio- [such as sn1 (16:0) or *sn*2 (18:1 (9Z)) for GPs] [89], positional (position of double bond), or geometric (cis/trans conformation of the double bond i.e. Z/E) isomers [90]. Typical example of glycerophospholipids is shown in Figure 2. Zandkarimi *et al*. (2019) separated, co-eluted plasmalogen phosphatidylethanolamine (PE p-) PE (p-36:1) and PE (p-38:2) lipids in mice brain tissue using LC-IM-MS. As both the PE had same retention time but were separated clearly in the ion mobility region with different drift time bin number [91]. Thus, structural elucidation was feasible due to IMS drift time, high collisional energy in transfer region and clear fragmentation pattern [76].

Technical developments in both hardware and software, empowers researchers to implement IM-MS into their analytical workflows. The four major augmentations in lipidomics are firstly, IM-MS crucially resolves isobaric species, thereby improves separation of lipids. Secondly, IM fragmentation improves the spectral clarity of product ion spectra. This is crucial in both lipid identification and structural elucidation, which are grueling task due to the isomeric nature of many lipid species. On the other hand, analysis of product ion spectra derived from untargeted fragmentation acquisitions remains challenging due to the required powerful processing tools. Thirdly, IM improves separation of isomeric lipids. Lastly, CCS values obtained from IM-MS analysis effectively increase confidence in lipid identification. Finally, IM-MS can be used to comprehend the conformational dynamics of a lipid system and offering a unique means of characterizing flexibility and folding mechanisms.

### MS Lipidomics Imaging and in situ

Lipidomics provide spatial information about the lipid composition in tissues - sort of molecular microscope [92]. There are several desorption ionizations tools and imaging MS techniques but ‘Lipidomics standard initiative’ (https://lipidomics-standards-initiative.org/) have recommended only secondary ion mass spectrometry (SIMS), desorption electrospray ionization (DESI) and matrix-assisted laser desorption/ionization (MALDI). Out of which, MALDI-imaging mass spectrometry (MALDI-IMS) is commonly used for lipid imaging in tissue sections.

#### MALDI-imaging mass spectrometry (MALDI-IMS)

MALDI-imaging mass spectrometry (MALDI-IMS) is a two-dimensional MALDI-MS technique which gives comprehensive profiles of molecular distributions of lipids [93] with high spatial resolution without extraction, purification, separation, or labeling of biological samples. This reveals the localization and abundance of hundreds of molecular species, especially lipids on the tissue slice in a single measurement thus helping in understanding the cellular profile of the biological system. Shimma *et al*. (2007) used this study the abnormal distribution of phospholipids in colon cancer liver metastasis [93].

This technique is widely used in brain and skin lipidomics as well [94]. Goto-Inoue *et al*. (2011) have used TLC-Blot-MALDI-IMS to study detailed structural analysis of lipids from human brain samples [95]. This was done by a three-step process, by running TLC once, transferring to a polyvinylidene difluoride membrane and finally detection by MALDI-IMS. Thus it was possible to separate, visualize and identify phosphatidylserine (PS) (diacyl-18:0/20:4), phosphatidylcholine (PC) (diacyl-16:0/18:1), and sphingomyelin (SM) (d18:1/C18:0) at *m/z* 812.5, 753.5 and 782.5 in gray matter. It was proposed that, this system would be useful in fully analyzing lipid compositions including minor components. Kendall *et al*. applied this technique in skin lipidomics [95] by analyzing *ex vivo* human skin cutaneous lipids for assessing alterations in lipid profiles linked to specific skin conditions. Both MALDI and DESI skin imaging are used for analysis of the whole skin sections, though the analysis time was same, disadvantages being, the former had matrix selection and later had less spatial resolution. SIMS imaging is known for high spatial resolution but a low mass range, which can decipher the spatial distribution of multiple lipids at subcellular levels. A completely new technique was studied using Infrared matrix assisted laser desorption electrospray ionization mass spectrometry (IR-MALDESI), combines many benefits of MALDI and ESI. IR-MALDESI works by combining laser desorption of neutrals with subsequent ionization by ESI to increase lipid abundance with great coverage relative to commonly used parameters in negative polarity [96]. This technique can be used for quantitative analysis and ideally for drug distribution studies [96].

The use of other ambient ionization tools, including rapid evaporative ionization mass spectrometry (REIMS) and direct analysis in real time (DART) allow rapid, real-time screenings of lipids for predictive, preventive, and personalized medicine. REIMS based methods require no preparative steps or time-consuming cell extractions which are discussed later in this review. IR-MALDESI sensitivity and selectivity was augmented using silver cationization of olefinic lipids in human serum using calcifediol as the reference [97].

#### Rapid Evaporative ionization mass spectrometry (REIMS)

REIMS is truly ambient analysis technique, for fast, easy molecular profiling with zero sample preparation. This is highly versatile technique as it can be used for both biological solid and liquid samples, as this provides quick determination of differences within and between samples. This is achieved by simple evaporation of the sample by Joule heating or laser irradiation, the aerosol generated is introduced orthogonally to the inlet of the mass spectrometer such as high performance ToF-MS. This technique has been used in intra-surgical tissue classiﬁcation [98], bacterial identiﬁcation [96], rapid proﬁling of cell lines [100] the analysis of plant material, food applications [101] and bioliquid samples. This technique has a potential to combine with other sampling techniques in providing a holistic profiling approach.

Few scientists have used matrix assisted version of the technique (MA-REIMS) to enhance the signal intensity in identifying strong phospholipid signal in the tumor which is absent in normal breast tissue [98]. In another group, in situ and real-time recognition model was employed in identifying 12 fatty acids and 37 phospholipids using “iKnife” and REIMS in discrimination of salmon and rainbow trout without sample preparation and adulteration of minced meats [102]. Despite so many advantages, the major setback of this technique is, it provides only moderate amounts of chemical information and repeatability, for further information we should still bank on LC-MS.

#### Direct analysis in real time-mass spectrometry (DART-MS)

DART-MS is an ambient pressure ionization technique enabling instantaneous and sensitive analyses of gases, liquids and solids [103]. It is based on the interactions of long lived electronic or vibronic excited-state molecule with sample and atmospheric gases at atmospheric pressure. This technique does not require laborious sample preparation, as ionization takes place directly on the sample surface, deposited or adsorbed on to surfaces or that are being desorbed into the atmosphere. The combination of this source with a high-resolution mass spectrometer (commercially available with JOEL as DART-Accu TOF) offers a rapid qualitative and quantitative measurements. It has numerous applications in the field of food science, forensics, and clinical analysis (https://www.jeol.co.jp/en/applications/pdf/ms/ms_note_en002.pdf). “No-prep” analysis of lipids in cooking oils and detection of adulterated olive oil is an application using DART-Accu TOF. Despite numerous advantages, DART ionization does have several inherent limitations. Firstly, fragmentation occurs at higher plasma temperatures, hindering spectra interpretation and accurate determination of the mass of intact molecules, well the decomposition fragments can also contribute to the structural information [104]. Secondly, this technique subjects the analyte to oxidation artifacts, owing to the design of the instrument i.e., distance from the capillary outlet [104]. Lastly, saturated hydrocarbons can undergo hydride abstraction hindering the quantitative analysis. For example, signals from aliphatic hydrocarbons and monounsaturated hydrocarbons are indistinguishable due to the same carbon length [105].

## Conclusions

In this review, we have outlined different analytical strategies for a comprehensive identification, complete structural characterization and accurate quantitation of biogenic lipid molecules with a fewer bottle necks. It must be noted, that each technique has its own strengths and weaknesses for example high mass resolution results in accurate mass but with natural limitations. Therefore, a mixture of analytical devices (chromatography, spectrometry, mass spectrometry and hyphenated methods) helps to cope up with the complexity of the lipids structure. However, lipidomics provide enormous data especially non-targeted lipids and it is critical to evaluate it using bioinformatics solutions [106]. The lipid information is available as web resources namely, cyberlipid center (http://www.cyberlipid.org/) and AOCS lipid library (http://lipidlibrary.aocs.org/). Tools that have been developed for analysis of MS-based lipidomic data include MS and MS/MS data by lipid consortium such as LIPID MAPS Lipidomics Gateway (www.lipidmaps.org/resources/tutorials/databases.html) and National Institute of Standards and Technology (NIST) (http://chemdata.nist.gov/); and commercialized software such as Lipidview™ (https://sciex.com/products/software/lipidview-software), LipidSearch™ (https://www.thermofisher.com/hr/en/home/industrial/mass-spectrometry/liquid-chromatography-mass-spectrometry-lc-ms/lc-ms-software.html), and SimLipid (www.premierbiosoft.com) also become available. This will undoubtedly be a valuable tool for investigation of many diseases, physiological processes or in disease biomarker discovery.

## Figures and Tables

**Table 1.** Examples of eight categories of lipids

Categories	Structures Examples	Typical Classes: Subclasses
Fatty acyls, FA	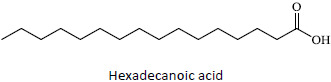	Fatty acids: Straight chain Fatty acids Eicosanoids Fatty alcohols Fatty esters Fatty amides
Prenol lipids, PR	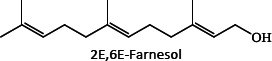	Isoprenoids Quinones and hydroquinones Polyprenols
Glycerolipids, GL	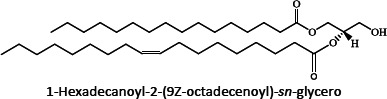	Monoradylglycerols: monoacyl glycerols Diradylglycerols: diacyl glycerols Triradylglycerols: triacyl glycerols
Glycerophos- pholipids, GP	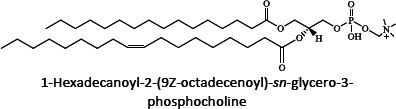	Glycerophosphocholines Glycerophosphoenolamines Glycerophosphoserines Glycerophospholycerols Glycerophosphoglycero-phosphates Glycerophosphoinositols Glycerophosphoglycerophospho-glycerols
Sphingolipids, SP	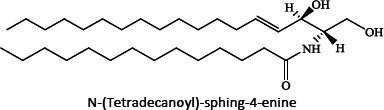	Sphingoid bases Ceramides Phosphosphingolipids Neutral glycosphingolipids Acidic glycosphingolipids
Saccharolipids, SL	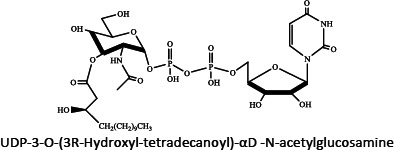	Acylaminosugars Acylaminosugar glycans Acyltrehaloses Acyltrehalose glycans
Sterol lipids, ST	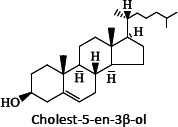	Sterols Cholesterol and derivatives Steroids Bile acids and derivatives
Polyketides, PK	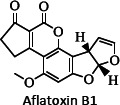	Macrolide polyketides Aromatic polyketides Nonribosomal peptide/ polyketides hybrids
